# Public Knowledge, Practice, and Attitude Regarding Cancer Screening: A Community-Based Study in Saudi Arabia

**DOI:** 10.3390/ijerph20021114

**Published:** 2023-01-08

**Authors:** Dalia Ahmed Elmaghraby, Ahmed Ali Alshalla, Anas Alyahyan, Muntathir Altaweel, Ahmad Mohammed Al ben Hamad, Khalid Mohammed Alhunfoosh, Mohammed F. AlJuwaysim, Duaa Jawad Aljumah, Mohammed Abdullah Albahrani

**Affiliations:** Department of Pharmacy Practice, College of Clinical Pharmacy, King Faisal University, Al Hofuf 31982, Saudi Arabia

**Keywords:** cancer screening, knowledge, practice, population, early detection, cancer prevention health behavior

## Abstract

(1) Background: Cancer screening tests discover cancer at early stages, even before symptoms appear. When abnormal tissues or a malignant mass is found early, treatment and cure rates are improved. In late stages, the cancer may have grown and metastasized. This can negatively affect cancer treatment and reduce the overall survival rate. Screening tests are performed when a person is asymptomatic. Public awareness about cancer screening is crucial for the success of cancer screening programs and for consequently decreasing the morbidity and mortality rate due to cancer. (2) Aim: Assess the knowledge and perception of the community regarding cancer screening in Saudi Arabia. (3) Methodology: A descriptive cross-sectional study targeting the general population of Saudi Arabia was conducted from January to June 2022. The data were collected using a structured validated electronic questionnaire. The study questionnaire covered participants’ personal data, medical history, source of data, and participants’ knowledge, attitude, and practice items. The questionnaire was used as a digital survey and was distributed electronically to the target population. (4) Results: A total of 1313 participants completed the study questionnaire. The participants’ ages ranged from 18 to 67 years, with a mean age of 28.3 ± 11.4 years old. Overall, 60.4% of the study participants knew about cancer screening. Regarding the benefits of cancer screening, 91.8% of the participants reported knowing that the early detection of cancer helps treatment, and 81.1% knew that the early detection of cancer improves treatment outcomes. Moreover, 441 (33.6%) of the participants had good knowledge regarding cancer and cancer screening, while 872 (66.4%) had poor levels of knowledge. Furthermore, 106 (8.1%) of the participants underwent cancer screening. (5) Conclusions: The study results revealed that participants’ awareness regarding cancer and cancer screening was low, especially for approaches to reduce cancer risk. Additionally, the study participants’ practice regarding cancer screening was low. The health care authority should plan for population-based efficacious cancer screening programs. In addition, cancer screening information and the benefits of early detection can be disseminated through social media to target the desired populations.

## 1. Introduction

Cancer is a leading cause of death in the United States and worldwide [1,2]. In 2018, in the United States, it was reported that nearly 1.7 million cancers were diagnosed in men and women, with a corresponding 609,000 cancer-related deaths [2]. In reference to the records that were published in September 2018, there were 16,210 (50 of 100,000 individuals) newly diagnosed individuals with cancer in Saudi Arabia in 2015 [3]. Many preventions and early detection methods were recommended to help reduce the prevalence of some types of cancer [4,5,6,7,8]. When abnormal tissue, hyperplasia, or cancer is found early, treatment or cure rates are improved. [9,10,11] Cancer screening modalities can help discover cancer at an earlier stage, even before the disease becomes symptomatic [12]. By the time symptoms appear, the cancer is advanced, and the patient enters the late stage. [13] This can negatively affect cancer treatment and reduce cure rates [11,13,14]. Hence, screening tests are performed when a person has no cancer symptoms [14]. Moreover, different studies have shown that early screening programs are cost-effective compared to not screening [15,16].

Cancer screening modalities should be cost-effective, non-invasive, and decrease the mortality rate by early detection of cancer [8]. Low-dose computed tomography (CT) is a screening test that is recommended for adults who have a high risk of developing lung cancer. This is based on their age and smoking history [11]. Screening mammograms are x-rays that are done on women who have no symptoms [5]. The goal of screening mammograms is to find cancer when it is still small enough that a woman or her doctor can not feel it. Finding small breast cancers early with a screening mammogram greatly decreases the mortality and morbidity due to cancer [5]. Several tests can be used to find polyps or cancer in the colon. These tests include stool tests, flexible sigmoidoscopy, colonoscopy, and CT colonography [6,13,15].

Public awareness of cancer screening is crucial for the success of cancer screening programs and for decreasing the mortality rate [13]. Assessing the community’s awareness regarding cancer screening could help the health authorities improve health screening programs and campaigns Because of this, the current study aimed to assess public knowledge, attitude, and practice regarding cancer and cancer screening in Saudi Arabia.

## 2. Materials and Methods

A descriptive cross-sectional study targeting the general population in Saudi Arabia was conducted from January to June 2022. The data were collected using a pre-structured electronic questionnaire initiated by the researchers after an intensive literature review and was validated by experts’ consultations to fulfill the purpose of this study and avoid errors in the data collection. A panel of 3 oncology experts revised the questionnaire to assess it and suggest any modifications. Ten participants were interviewed personally to check the clarity of the questions. The study was conducted in accordance with the Declaration of Helsinki and approved by the Ethics Committee of King Faisal University (protocol code KFU-REC-2022-JAN-EA000383). The study questionnaire covered participants’ personal data, including age, gender, educational level, work and monthly income, persona, and medical history of cancer. The second section included participants’ knowledge regarding cancer and screening methods and the source of this knowledge. The last section included a question focused on participants’ practices and attitudes towards screening, reasons for undergoing cancer screening, and the reasons for not undergoing cancer screening. Additionally, participants’ attitudes regarding the accessibility of screening guidelines and the need for more information was included. The questionnaire was used as a digital survey and distributed to all of the participants in a private and anonymous manner. The question was designed to elicit information in a concise and objective manner. Furthermore, a logical layout was used in the questions so the subsequent answer would be based on the prior responses. The final questionnaire was uploaded and distributed through social media platforms.

### 2.1. Sample Size

We used the Raosoft^®^ sample size calculator with a margin of error of 5% and a confidence level of 95%, thus the proposed sample is 385, and we obtained 1313 responses.

The sample size n and margin of error E are given by:

x = Z(c/100)2r(100 − r)

n = Nx/((N − 1)E2 + x)

E = Sqrt[(N − n)x/n(N − 1)]

where N is the population size, r is the fraction of responses, and Z(c/100) is the critical value for the confidence level c.

### 2.2. Data Analysis

After the data were extracted, they were adjusted, coded, and fed into the statistical software IBM SPSS version 22 (SPSS, Inc. Chicago, IL, USA). All of the statistical analyses were conducted using two-sided tests. Statistical significance was considered achieved when the *p*-value was less than 0.05. Regarding the knowledge and awareness domains, each correct choice scored one point, and the total summation of all scores of the different items was calculated. A participant with a total score of less than 60% was considered to have poor knowledge, while good knowledge was considered if they had a score of 60% or more of the total. A descriptive analysis based on the frequency and percent distribution was undertaken for all of the variables, including participants’ personal data, medical and family history, and the sources of their information. Additionally, participants’ knowledge and awareness regarding cancer and cancer screening were described in frequency tables and were graphed. Additionally, participants’ perceptions and practices regarding cancer and cancer screening were also tabulated and graphed. Crosstabulation was used to assess the factors associated with public knowledge regarding cancer and cancer screening. The relationships were tested using the Pearson chi-square test and the Exact probability test for small frequency distributions.

## 3. Results

### 3.1. Participants’ Characteristics

A total of 1313 participants completed the study questionnaire. The age of the participants ranged from 18 to 67 years, with a mean age of 28.3 ± 11.4 years old. Five hundred eighty-five (44.6%) participants were males, 818 (62.3%) were single, and 437 (33.3%) were married. Regarding education, 937 (71.4%) participants were university graduates, and 340 (25.9%) had a secondary school level of education. A total of 249 (19%) were healthcare workers. A monthly income of less than 9000 SR was reported among 281 (21.4%), and 361 (27.5%) had a monthly income of 9000–15,000 SR. Twenty-six (2%) had a personal history of cancer, 578 (44%) had a family history of cancer, and 208 (15.8%) had a chronic health problem (Table 1).

### 3.2. Public Knowledge Regarding Cancer and Cancer Screening, Saudi Arabia

Overall, 60.4% of the study participants knew about cancer screening. Regarding the benefits of cancer screening, 91.8% reported that detecting cancer early aids treatment, 81.1% knew that the early detection of cancer improves treatment outcomes, 72.6% stated that individuals with a family history of cancer need cancer screening, and 45.4% knew that some types of cancer could be avoided. As for the types of cancer that can be detected early through screening, 93.9% knew about breast cancer, 29.9% reported colon cancer, 27.5% identified prostate cancer, and 12.9% knew about anal cancer. Overall, 11.2% of the study participants thought that they had good knowledge of how to reduce cancer risk, while 14.8% reported having moderate knowledge, and 32.1% evaluated their knowledge as poor. When asked about the factors that may reduce the risk of cancer, the participants’ answers were smoking cessation (83%), increased physical activity (79.2%), consuming a healthy diet (75.9%), and avoiding environmental pollutants (67.4%) (Table 2).

**Figure 1:**
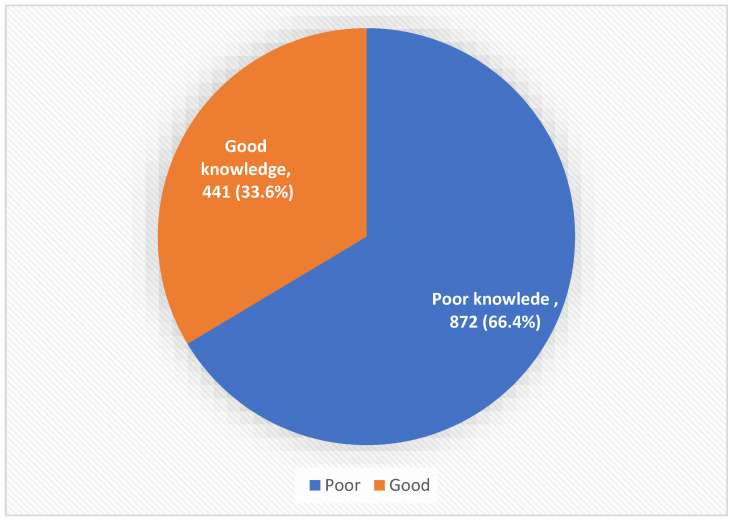
Overall public knowledge level regarding cancer and cancer screening, Saudi Arabia.

Four hundred forty-one (33.6%) participants had good knowledge regarding cancer and cancer screening, while 872 (66.4%) had poor knowledge levels (see Figure 1).

### 3.3. Public Practice Regarding Cancer Screening, Saudi Arabia

One hundred six of the study participants (8.1%) underwent cancer screening. The reasons for cancer screening were related to the early detection of cancer (53.8%), following the Saudi Ministry of Health’s recommendations (52.9%), and having a family history of cancer (38.5%). A total of 71 (67%) underwent screening at a primary health care center or hospital, while 33 (31.1%) participated in a screening campaign. A total of 83 (78.3%) underwent cancer screening for 1–4 years and 10 (9.4%) for 5–10 years. Among those who did not experience screening, the reasons for that were lack of cancer symptoms (77.5%), still being young (31.5%), lack of time (23%), fear of the screening results (18.3%), and fear of the screening procedure (15.5%) (Table 3).

**Figure 2:**
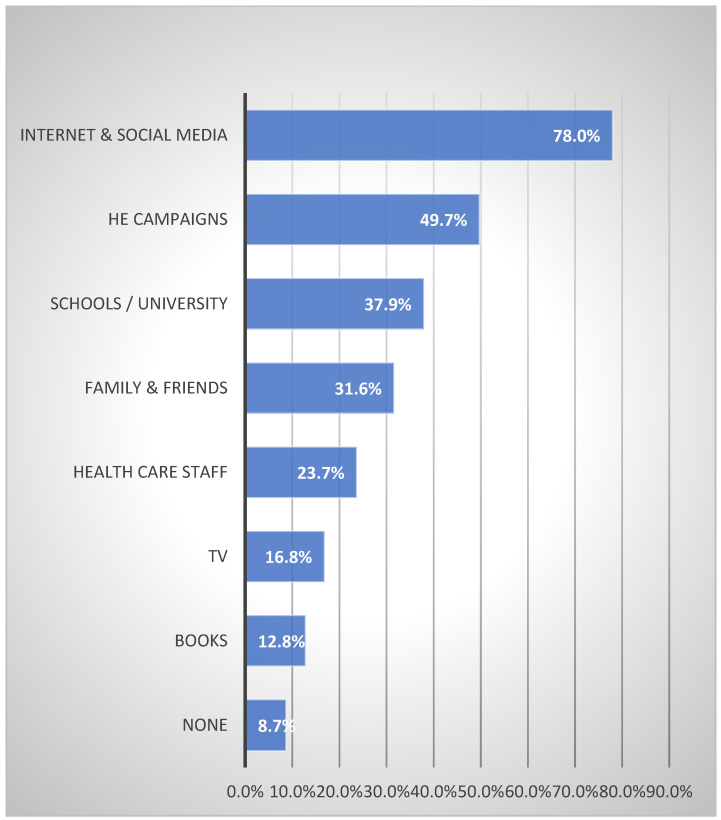
Source of information regarding cancer and screening among the study population.

Figure 2 were 78% internet and social media, 49.7% health campaigns, 37.9% undergraduate courses, 31.6% family and friends, and 23.7% healthcare staff.

### 3.4. Participants’ Attitudes towards Cancer Screening, Saudi Arabia

**Figure 3:**
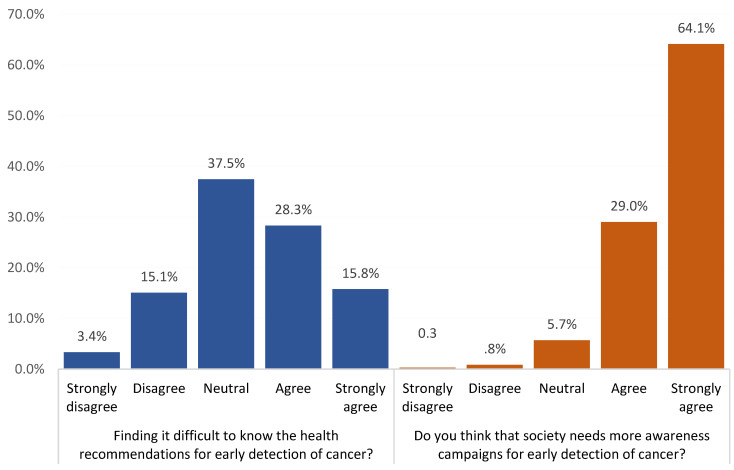
Participants’ attitudes towards cancer screening, Saudi Arabia.

Overall, 44.1% of the participants agreed that they found it difficult to know the health recommendations for the early detection of cancer. Additionally, 93.1% think that society needs more awareness campaigns for the early detection of cancer (Figure 3).

### 3.5. Factors Associated with Public Knowledge Regarding Cancer and Cancer Screening, Saudi Arabia

Good knowledge was detected among 37.7% of the participants aged 20–29 years versus 28.5% of others under 20 years old (*p* = 0.025). Additionally, 58.2% of the healthcare workers had good knowledge compared to 27.8% of the others (*p* = 0.001). Moreover, 38.2% of those with a family history of cancer had a good knowledge level in comparison to 29.1% of those without a family history of cancer (*p* = 0.004). In addition, 40.9% of those with chronic health problems had good knowledge versus 32.2% of those without chronic health issues (*p* = 0.015). Good knowledge was detected among 45.3% of those who experienced cancer screening (*p* = 0.008) (Table 4.).

## 4. Discussion

Cancer screening is a significant approach to cancer prevention, and it depends mainly on the public willingness to participate in screening campaigns [17,18,19,20]. Unfortunately, the reported participation rate of the population in such campaigns is low and consequently affects the morbidity and mortality rates due to cancer [21,22]. Thus, this study aimed to analyze the public attitude and perception toward cancer screening in Saudi Arabia.

As for knowledge, the study showed that nearly one-third of the participants had a good knowledge level regarding cancer and cancer screening. Comparable results were published in a review of 19 articles regarding cancer screening, which showed that the overall knowledge of cervical cancer among women was 40.22% [23]. Moreover, a cross-sectional study in a primary care center in Riyadh showed that the participants had inadequate knowledge of colorectal cancer screening [24]. Conversely, a study conducted in Hong Kong showed that elderly males had good knowledge and attitudes toward colorectal cancer and its screening [25]. In Madinah, Saudi Arabia, Jarb AF et al. [26] estimated that 77% of the contributors had heard about prostate cancer, and 52.5% had heard about its screening tests. They revealed that approximately 10.6% of all of the contributors had good knowledge, 41.9% had fair knowledge, and 47.5% had poor knowledge. Only 3.9% of the participants underwent the prostate-specific antigen test. Similarly, in Riyadh city, a study revealed that the knowledge of prostate cancer was poor among the male participants [27]. These findings are concordant with other studies conducted in Saudi Arabia, Egypt, and Jordan that reported inadequate knowledge and a fair attitude toward cancer examination and screening practices [28].

As for the practice of cancer screening, the current study showed that only 8.1% had been subjected to cancer screening. The reasons behind their screening were early detection of cancer (53.8%), following the recommendations of the Saudi Ministry of Health (52.9%), and having a family history of cancer (38.5%). The main screening site reported by one-third of the study participants (31.1%) was the screening campaign. Among those who had not been subjected to a screening, the most reported reasons were a lack of cancer symptoms (77.5%), still being young (31.5%), a lack of time (23%), fear of the screening results (18.3%), and fear of the screening procedure (15.5%). Many previous studies have reported on the reasons for undergoing screening and the reasons for not participating in screening. For instance, Paskett ED et al. found that 67% of all women claimed that physicians did not consult them about mammograms, although 75% had a regular check-up in the last year [29]. In addition, In Saudi Arabia, a study revealed that 55.3% were willing to undergo a colonoscopy or sigmoidoscopy. Conversely, among the group that did not agree to undergo screening, 77.4% of them would undergo non-invasive procedures such as radiological screening using barium enema and/or a computed tomography scan of the abdomen [30]. In 2013, the Saudi health interview survey revealed that nearly 89% of women did not have a clinical breast examination in the past year, and 92% had never had a mammogram [31]. It is worth noting that cancer screening is offered for free in Saudi Arabia for the whole population. In contrast, a retrospective study analyzed the screening pattern of different cancer types in the United States and reported that the reduced use of cancer screening is attributed to a lack of contact with a doctor, regular healthcare facilities, and no insurance allowance [32].

In this study, we analyzed the factors associated with obtaining better knowledge. Population awareness and knowledge were significantly higher among healthcare staff due to the nature of their work being related to the study field. In addition, those with a family history of cancer had better knowledge, which could be attributed to the information they get from the oncologist during hospital visits or because they may search the internet for their relatives’ clinical condition. Moreover, participants with chronic health problems had good knowledge regarding cancer screening, which could be explained by their frequent visits to hospitals and other healthcare facilities. The participants of the age group 20–29 years old had significantly better knowledge than other age groups. This could be attributed to the more frequent use of social media and the internet

Similarly, the Saudi health interview survey reported that women with hypertension and well-educated women were more likely to participate in breast cancer screening [31]. Similarly, the national health survey reported that some chronic diseases were associated with higher cervical and breast cancer screening among women in France [33].

Our study has some limitations. Initially, all of the selected variables are self-reported and may be subject to recall bias. Second, the study participants are of a young age, and this may be attributed to the fact that the majority of the Saudi population is young [34]. However, our survey covered all regions of Saudi Arabia, asked about different types of cancer, and targeted males and females.

Our results showed that the majority of the participants’ information comes from the internet and social media. This emphasizes the importance of online educational programs targeting the population. Similarly, internet-based applications and online health programs are effective in different diseases assessments and treatments [35,36,37].

The World Health Organization (WHO) considers the early detection of cancer through public education as the first pillar in decreasing breast cancer mortality [38]. In the current study, the participants were asked about the cancer types that could be screened; most of the participants (93.9%) selected breast cancer, while 29.9% picked colon cancer, 27.5% selected prostate cancer, and 22.8% knew that lung cancer could be detected early. According to the National Comprehensive Cancer Network (NCCN) guidelines, high-risk individuals should be screened for lung cancer at the age of 50 by using low-dose computed tomography (LDCT) of the chest [39]. In addition, breast cancer screening is performed for women at the age of 40 by mammogram [40]. Additionally, NCCN recommends screening for colorectal cancer by using either stool/fecal-based tests, colonoscopy, flexible sigmoidoscopy, or CT colonography starting at age 50 for average-risk persons [41]. Moreover, the NCCN recommended prostate cancer for men aged 45–70 years using prostate-specific antigen and digital rectal exam [17].

## 5. Conclusions

The study revealed that public awareness regarding cancer and cancer screening was low, particularly for approaches to reduce cancer risks. Awareness was significantly higher among the participants with chronic diseases and those with a family history of cancer. Additionally, the study revealed that participant practices regarding cancer screening were poor and require urgent intervention. The health care authority should plan for population-based efficacious cancer screening programs. In addition, cancer screening information and the benefits of early detection could be disseminated through social media to target the desired population.

## Figures and Tables

**Table 1:** Bio-demographic data of study participants, Saudi Arabia.

Bio-Demographic Data	No	%
Age in years		
*<20*	295	22.5%
*20–29*	594	45.2%
*30–39*	156	11.9%
*40+*	268	20.4%
Gender		
*Male*	585	44.6%
*Female*	728	55.4%
Education		
*Below secondary*	36	2.7%
*Secondary*	340	25.9%
*University/postgraduate*	937	71.4%
Work in a healthcare sector		
*Yes*	249	19.0%
*No*	1064	81.0%
Monthly income		
*<9000 SR*	281	21.4%
*9000–15,000 SR*	361	27.5%
*16,000–25,000 SR*	200	15.2%
*>25,000 SR*	107	8.1%
*Preferer not to mention*	364	27.7%
Marital status		
*Single*	818	62.3%
*Married*	437	33.3%
*Divorced/widow*	58	4.4%
Personal history of cancer		
*Yes*	26	2.0%
*No*	1287	98.0%
Family history of cancer		
*Yes*	578	44.0%
*No*	616	46.9%
*Don’t know*	119	9.1%
Presence of chronic health problems		
*Yes*	208	15.8%
*No*	1105	84.2%

**Table 2:** Public knowledge regarding cancer and cancer screening, in Saudi Arabia.

Knowledge Items	No	%
Do you know what is cancer screening?		
*Yes*	793	60.4%
*No*	520	39.6%
Benefits of cancer screening		
*Detecting cancer early helps treat it*	1205	91.8%
*Early detection of cancer improves treatment outcomes*	1065	81.1%
*Individuals with a family history of cancer need cancer screening*	953	72.6%
*Some types of cancer we can avoid*	596	45.4%
*There is no type of cancer that we can avoid*	78	5.9%
*No benefit for cancer screening*	13	1.0%
Which types of cancer can be screened?		
*Breast cancer*	1233	93.9%
*Cancer colon*	392	29.9%
*Anal cancer*	169	12.9%
*Prostate cancer*	361	27.5%
*Lung cancer*	300	22.8%
*Ovarian cancer*	271	20.6%
*Blood cancer*	356	27.1%
*LN cancer*	241	18.4%
*Brain cancer*	178	13.6%
*Bone cancer*	160	12.2%
How do you assess your knowledge about cancer risk?		
*Good knowledge*	147	11.2%
*Moderate knowledge*	194	14.8%
*Poor knowledge*	421	32.1%
*Not sure of my knowledge*	551	42.0%
Factors that reduce the risk of cancer?		
*healthy diet*	996	75.9%
*Exercising and increasing physical activity*	1040	79.2%
*Decrease exposure to environmental pollutants*	885	67.4%
*Smoking cessation*	1090	83.0%
*Screening for people with a family history of cancer*	3	0.2%
*Consuming vitamins*	348	26.5%
*Others*	17	1.3%

**Table 3:** Public practice regarding cancer screening, Saudi Arabia.

Practice	No	%
Previously experienced cancer screening		
*Yes*	106	8.1%
*No*	1207	91.9%
Causes for undergoing cancer screening		
*Following the recommendation of the Saudi Ministry of Health*	55	52.9%
*For early detection of cancer*	56	53.8%
*Had a family history of cancer*	40	38.5%
Where did you experience cancer screening?		
*Primary healthcare center/hospital*	71	67.0%
*Screening campaigns*	33	31.1%
*Self-screening*	2	1.9%
Time since last screening?		
*<1 year*	9	8.5%
*1–4 years*	83	78.3%
*5–10 years*	10	9.4%
*>10 years*	4	3.8%
Causes of not undergoing cancer screening		
*Had no symptoms*	936	77.5%
*Still young*	380	31.5%
*Lack of time*	278	23.0%
*Fear of screening results*	221	18.3%
*Fear of screening procedure*	187	15.5%
*Financial difficulty*	159	13.2%
*No benefit to do*	119	9.9%
*Don’t know screening settings*	19	1.6%
*Others*	19	1.6%

**Table 4:** Factors associated with public knowledge regarding cancer and cancer screening, Saudi Arabia.

Factors		Overall Knowledge Level				*p*-Value
		Poor		Good		
		No	%	No	%	
Age in years	<20	211	71.5%	84	28.5%	0.025 *
	20–29	370	62.3%	224	37.7%	
	30–39	104	66.7%	52	33.3%	
	40+	187	69.8%	81	30.2%	
Gender	Male	386	66.0%	199	34.0%	0.767
	Female	486	66.8%	242	33.2%	
Education	Below secondary	26	72.2%	10	27.8%	0.061
	Secondary	242	71.2%	98	28.8%	
	University/above	604	64.5%	333	35.5%	
Work at healthcare sector	Yes	104	41.8%	145	58.2%	0.001 *
	No	768	72.2%	296	27.8%	
Marital status	Single	540	66.0%	278	34.0%	0.763
	Married	291	66.6%	146	33.4%	
	Divorced/widow	41	70.7%	17	29.3%	
Personal history of cancer	Yes	17	65.4%	9	34.6%	0.911
	No	855	66.4%	432	33.6%	
Family history of cancer	Yes	357	61.8%	221	38.2%	0.004 *
	No	437	70.9%	179	29.1%	
	Do not know	78	65.5%	41	34.5%	
Had chronic health problems	Yes	123	59.1%	85	40.9%	0.015 *
	No	749	67.8%	356	32.2%	
Previously experienced cancer screening	Yes	58	54.7%	48	45.3%	0.008 *
	No	814	67.4%	393	32.6%	
Source of information	Internet & social media	668	65.2%	356	34.8%	0.001 * ^$^
	health campaigns	394	60.3%	259	39.7%	
	Health care staff	144	46.3%	167	53.7%	
	Schools/university	277	55.6%	221	44.4%	
	Family & friends	265	63.9%	150	36.1%	
	Books	76	45.2%	92	54.8%	
	TV	127	57.5%	94	42.5%	
	None	93	81.6%	21	18.4%	

*p*: Pearson X^2^ test. ^$^: Exact probability test. * *p* < 0.05 (significant).

## Data Availability

All data are available in the study.
